# Original Department of This Journal

**Published:** 1879-03

**Authors:** 


### Original Department of this Number.

On account of the liberality of our friends in original contributions, we have excluded the usual miscellaneous selections, and offer our readers an entirely Original Journal. Book reviews are also from necessity postponed for next issue, and mention of Pamphlets Received.

